# A multicenter, open-label, long-term safety and tolerability study of DFN-02, an intranasal spray of sumatriptan 10 mg plus permeation enhancer DDM, for the acute treatment of episodic migraine

**DOI:** 10.1186/s10194-017-0740-3

**Published:** 2017-03-01

**Authors:** Sagar Munjal, Elimor Brand-Schieber, Kent Allenby, Egilius L.H. Spierings, Roger K. Cady, Alan M. Rapoport

**Affiliations:** 1 Dr. Reddy’s Laboratories Ltd., 107 College Road East Princeton, Princeton, NJ 08540 USA; 2 Dental Medicine Headache & Face Pain Program Tufts Medical Center, Craniofacial Pain Center Tufts University School, 800 Washington Street Boston, Boston, MA 02111 USA; 3 Clinvest/A Division of Banyan Inc., 3805 S Kansas Expy Springfield, Springfield, MO 65807 USA; 4 David Geffen School of Medicine at UCLA, Los Angeles, CA USA

**Keywords:** Episodic migraine, Acute treatment, Intranasal sumatriptan, DDM, Sumatriptan, Long-term safety

## Abstract

**Background:**

DFN-02 is a novel intranasal spray formulation composed of sumatriptan 10 mg and a permeation-enhancing excipient comprised of 0.2% 1-O-n-Dodecyl-β-D-Maltopyranoside (DDM). This composition of DFN-02 allows sumatriptan to be rapidly absorbed into the systemic circulation and exhibit pharmacokinetics comparable to subcutaneously administered sumatriptan. Rapid rate of absorption is suggested to be important for optimal efficacy. The objective of this study was to evaluate the safety and tolerability of DFN-02 (10 mg) in the acute treatment of episodic migraine with and without aura over a 6-month period based on the incidence of treatment-emergent adverse events and the evaluation of results of clinical laboratory tests, vital signs, physical examination, and electrocardiograms.

**Methods:**

This was a multi-center, open-label, repeat-dose safety study in adults with episodic migraine with and without aura. Subjects diagnosed with migraine with or without aura according to the criteria set forth in the International Classification of Headache Disorders, 2nd edition, who experienced 2 to 6 attacks per month with fewer than 15 headache days per month and at least 48 headache-free hours between attacks, used DFN-02 to treat their migraine attacks acutely over the course of 6 months.

**Results:**

A total of 173 subjects was enrolled, 167 (96.5%) subjects used at least 1 dose of study medication and were evaluable for safety, and 134 (77.5%) subjects completed the 6-month study. A total of 2211 migraine attacks was reported, and 3292 doses of DFN-02 were administered; mean per subject monthly use of DFN-02 was 3.6 doses. Adverse events were those expected for triptans, as well as for nasally administered compounds. No new safety signals emerged. Dysgeusia and application site pain were the most commonly reported treatment-emergent adverse events over 6 months (21% and 30.5%, respectively). Most of the treatment-emergent adverse events were mild. There were 5 serious adverse events, all considered unrelated to the study medication; the early discontinuation rate was 22.5% over the 6-month treatment period.

**Conclusion:**

DFN-02 was shown to be well tolerated when used over 6 months to treat episodic migraine acutely.

## Background

In the acute treatment of episodic migraine (ie, patients with fewer than 15 headache days per month [1]), 5-HT_1B1D_ agonists (triptans) are recommended as first-line therapy for moderate and severe attacks and for mild attacks that have been unresponsive to analgesics or nonsteroidal anti-inflammatory drugs in the past [2]. Oral sumatriptan, the most widely used drug in the triptan class [3–5], is effective and safe [6], but it may not be an optimal choice for many attacks. Its poor bioavailability (15%) and slow absorption (T_max_ ≥ 2 h) lead to a slow onset of action (45–60 min) [7], an issue exacerbated in many patients by migraine-associated gastric stasis [8–10]. To varying degrees, poor absorption and relatively delayed onset of action limit the clinical utility of all oral triptans [4, 6].

For attacks associated with nausea or vomiting, as well as in patients who cannot tolerate or experience dysphagia with oral triptans, evidence-based guidelines recommend the use of non-oral migraine-specific formulations [2]. Currently available alternatives, however, are of limited clinical utility in many patients. Subcutaneous (SC) sumatriptan 6 mg has good efficacy (60% pain-free at 2 h) and a fast onset of action (10 min post-dose) [11], but most SC sumatriptan-treated patients (59%) have injection site reactions, nearly half (42%) experience atypical sensations (eg, tingling, warm/hot, tightness/pressure [12]), and many migraineurs are averse to using injectable formulations [13, 14]. Transdermal sumatriptan, which has been linked with burns and scars at the application site [15] and has very limited efficacy (18% pain-free at 2 h [16]), has been voluntarily taken off the market. With the commercial formulation of sumatriptan intranasal spray (Imitrex® Nasal Spray, Imigran® Nasal Spray, GlaxoSmithKline), because the drug is not well absorbed through the nasal mucosa [17] (T_max_ 0.88–1.75 h) [18]), its onset of action (30 – 45 min) is only slightly faster than oral sumatriptan [7]. Poor absorption is also a problem with intranasal zolmitriptan (T_max_ ~3 h) [19], and sumatriptan nasal powder has reported a T_max_ of 20 min to as long as 2 h [20, 21]. These important clinical limitations, particularly in light of the attributes of acute treatment that are most important to migraineurs (rapid onset of action, complete relief, no recurrence, lack of side effects [22–24]) highlight a gap in migraine pharmacotherapy and emphasize the unmet need for a safe and highly effective non-oral migraine medication with a rapid onset of action.

DFN-02 — sumatriptan 10 mg plus 0.20% 1-O-n-Dodecyl-β-D-Maltopyranoside (DDM), a permeation-enhancing excipient — is a new intranasal migraine treatment under clinical development that is designed to overcome many of the limitations to currently available acute medications. Sumatriptan is a well-known headache medicine that has been extensively studied and described [4, 5, 25]. DDM, on the other hand, belongs to a class of surfactants known as alkylglycosides, which are non-ionic and metabolized to simple carbohydrates and alcohols or acids that have shown promise as permeation enhancers for intranasal medication [26–28]. DDM appears to loosen cell-cell junctions and enhance paracellular movement through the nasal epithelium by altering mucosal viscosity and membrane fluidity, increasing blood flow, and inhibiting ciliary beat frequency and drug metabolizing enzymes [28–32].

In a pharmacokinetic (PK) study in healthy subjects, DFN-02 had a markedly more rapid sumatriptan absorption profile than commercial intranasal sumatriptan, systemic exposure was similar to 4 mg SC sumatriptan, and plasma sumatriptan concentration peaked 5 min earlier than 4 mg SC and 6 mg SC [33] — results that suggest it may have efficacy comparable to a 4-mg SC dose of sumatriptan, which has not yet been demonstrated in migraine patients. With efficacy expected based on PK equivalency data, the sole objective of this study was to evaluate the safety and tolerability of DFN-02’s unique formulation in the acute treatment of migraine over a 6-month period, based on treatment-emergent adverse events (TEAEs), clinical laboratory results, and electrocardiograms.

## Methods

This was a multi-center, open-label, repeat-dose safety study in adults with episodic migraine with and without aura. The protocol, the patient information and consent form, and other relevant study documentation were approved by the Institutional Review Board (IRB) for each study center before initiation of the study. Protocol amendments were approved by the IRB before implementation or submitted to the IRB for information, as required. The study followed the Guidelines of the World Medical Association Declaration of Helsinki in its revised edition (Brazil, 2013), The International Council for Harmonisation guidelines for current Good Clinical Practice, the United States Food and Drug Administration Code of Federal Regulations, and the demands of national drug and data protection laws and other applicable regulatory requirements. Investigators and study staff recruited patients at the individual study sites; written consent to participate was provided after having been informed about the nature and purpose of the study, participation/termination conditions, and risks and benefits of treatment. The study was registered with ClinicalTrials.gov (NCT02279082).

### Subjects

To be enrolled in the study, subjects had to be adults aged 18 – 65 years diagnosed with acute migraine according to the criteria set forth in the International Classification of Headache Disorders, 2nd edition (ICHD-2) [34]. They also had to have a history of 2 – 6 attacks with or without visual aura per month, with 14 or fewer headache days monthly and at least 48 h of headache-free time between attacks; when aura was present, it could not have lasted longer than 60 min. Females of childbearing potential had to have a negative urine pregnancy test, not be lactating, and agree to practice a reliable form of contraception or abstinence during the study. Males (with female partners) agreed to practice a reliable form of contraception or abstinence during the study. Subjects also had to be willing and able to return to the study site within 72 h of the first use of study medication; record each attack and each instance of the use of DFN-02 and rescue medication in a patient paper diary for the duration of the study; provide written informed consent; and use the DFN-02 intranasal spray device correctly after instruction.

Subjects were excluded if they had medication overuse headache (as defined by ICHD-2); used any botulinum toxin treatment within 180 days of screening; changed dosages of migraine preventive medications during the 30 days before and through screening; took mini-prophylaxis for menstrual migraine; had hemiplegic migraine or migraine with brain stem aura or other forms of neurologically complicated migraine; had a history of stroke, transient ischemic attack, migralepsy, seizure disorder, ischemic coronary artery disease, Wolff-Parkinson-White syndrome or arrhythmias associated with other cardiac accessory conduction pathway disorders, uncontrolled hypertension, peripheral vascular disease, ischemic bowel disease, neurological or psychiatric impairment, or cognitive dysfunction. They were also ineligible if they could not differentiate between a migraine attack and a tension-type or cluster headache; were intolerant to any formulation of sumatriptan or had experienced a significant adverse event related to any triptan; had a history of nonresponse to 2 or more triptans.

Subjects were also disqualified for any of the following: taking medications or having an illness likely to affect the physiology of the nasal mucosa; abnormal nasal physiology or pathology; intolerance to nasal sprays; severe renal impairment; serum total bilirubin >  2.0 mg/dL; serum aspartate aminotransferase, alanine aminotransferase, or alkaline phosphatase >  2.5 times the upper limit of normal; 1-year history of alcohol or substance abuse; positive urine drug screen for illicit drugs or unexplained prescription drugs; received treatment with an investigational drug or device within 4 weeks of the screening visit or participated in a central nervous system clinical trial in the 3 months before screening; tested positive for human immunodeficiency virus, hepatitis B surface antigen, or hepatitis C virus; any medical condition that would have confounded the objectives of the study.

### Treatment

DFN-02 was provided in a single-use intranasal spray device designed to deliver 100 μL/spray containing 10 mg of sumatriptan plus 0.20% DDM.

### Study conduct

The study involved 9 visits: screening (Day -21 to Day -1), enrollment (Day 0), initial follow up (within 72 h of treatment), and 6 monthly visits (every 30 ± 3 days). At the screening visit, subjects signed informed consent and underwent the following assessments: inclusion/exclusion criteria, demographics, medical history, physical examination, vital signs, serology, urine pregnancy test, clinical labs, urinalysis, urine drug test, 12-lead electrocardiogram (ECG), adverse event and concomitant medications review, and device training and medication instruction. Hematology, clinical chemistry, urinalysis, urine drug screen, and human immunodeficiency virus, Hepatitis B surface antigen, and Hepatitis C virus antibody analyses were performed at a central laboratory (ACM Global Central Laboratory, US, 160 Elmgrove Park, Rochester, NY 14624). At each visit, 3 ECG readings were collected, no less than 5 min apart, and reviewed initially by the investigator for any immediate concerns, and then by a central reader (ERT, 1818 Market St., Suite 1000, Philadelphia, PA 19103).

Subjects were observed for 21 days after the screening visit to evaluate whether they satisfied inclusion criteria and had no medication overuse headache. The screening period was shortened up to Day -1 if subjects met inclusion criteria before this time. Subjects continued to take their normal migraine medication during the screening period.

At enrollment, screening assessments (with the exception of demographics) were re-performed; DFN-02 devices (individually blister packed and labeled), an Instructions for Use document, and a paper diary were dispensed; and rescue medication was determined. The quantity of DFN-02 devices initially dispensed (6 or 12) was determined by the frequency of attacks (≤ 3/month  =  6 devices; ≥ 4/month  =  12 devices); at subsequent resupply visits, unused devices were counted and additional devices were dispensed as needed. Throughout the study, additional instructions on the dispensation of the study medication were available through an interactive web-response system.

For the next 6 months, DFN-02 was self-administered once into 1 nostril (either right or left per subject’s choice) at the onset of acute migraine pain; for attacks with aura, subjects were instructed to use DFN-02 at the onset of pain, not at the onset of aura. If pain relief at 1 h post-dose was insufficient, subjects were permitted to take either another dose of DFN-02 or rescue medication. If a second dose of DFN-02 was taken and relief was still inadequate after 2 h, rescue medication was permitted; the rescue medication was chosen with the investigator at screening and adjusted as necessary. No more than 2 doses of DFN-02 were permitted in a 24-h period.

During the treatment period, subjects recorded the migraine pain start and stop for each attack (date and time), use of DFN-02 (date and time), and use of rescue medication (date, time, name, and dose) in the paper diary dispensed to them at each study visit. The paper diary was reviewed by investigators, and its data were entered into the electronic case report form by study site personnel.

### Assessments

There were no efficacy variables in this study. Safety variables included the incidence of AEs, clinical laboratory data (hematology, chemistry, and urinalysis), vital sign measurements (sitting blood pressure, pulse, respiration rate, and body temperature), physical examination findings, 12-lead ECG readings, urine pregnancy test results, study medication use, and concomitant medication use. The primary endpoint was the incidence of TEAEs during the 6-month treatment period based on the incidence of TEAEs, clinical laboratory results, and ECG findings. The number of study medication doses taken within a migraine attack was determined based on dosing and migraine start/end dates and times recorded by the subjects; values were corroborated by the site staff. These data were also reconciled with data in the Drug Accountability Log, which recorded all used and unused study medication.

Adverse events were coded by using the Medical Dictionary for Regulatory Activities (MedDRA, version 17.0) and classified by severity (mild, moderate, severe) and causality (not related, possibly related, probably related, definitely related). Treatment-emergent AEs were defined as AEs with a start date on or after the initial dose and up to 5 days after the last dose of DFN-02 or events that became worse in severity on or after the date of the first DFN-02 dose. Severe AEs were defined as AEs that prevented normal everyday activities and usually needed treatment or other intervention. Investigators also characterized the seriousness of AEs, and serious AEs (SAE) were defined as any untoward medical occurrences or effects that, at any dose, resulted in death or were life-threatening; required or prolonged inpatient hospitalization, resulted in persistent or significant disability/incapacity, or were congenital anomaly/birth defects. Severe AEs were not necessarily SAEs.

### Statistics

All analyses and summaries were produced using SAS version 9.3 or above (SAS Institute, Cary, NC). The study populations for analysis included all subjects who were screened (for disposition) and all subjects who received at least 1 dose of study medication (for all safety endpoints). Continuous variables were summarized using the number of observations (n), mean, standard deviation (SD), median, minimum, and maximum. Categorical variables were summarized using frequency and percentages of subjects. If there were multiple valid results at a given visit, the first value at each visit window was used, unless a test was repeated and the result suggested that the initial value was an error, in which case the last observation within the visit window was used.

Unless otherwise specified, baseline assessment was the latest available valid measurement taken prior to the initial dose administration of study medication, generally Day 0. Missing safety data were not imputed in this study.

No formal sample size calculation was performed. It was estimated that at least 150 subjects would need to be enrolled to ensure that at least 100 subjects completed the 6-month study period.

## Results

Twenty-five US investigator sites participated in the study. The first subject was enrolled into the study on 06 October 2014, and the last subject completed the study on 19 August 2015.

### Subjects

**Fig. 1:**
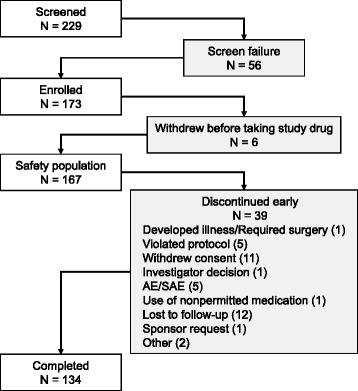
Disposition of subjects

A total of 229 subjects was screened, 173 subjects met inclusion criteria, 167 subjects received at least 1 dose of study drug and were evaluable for safety (96.5%), 134 subjects (77.5%) completed the 6-month treatment period, and 39 subjects (22.5%) discontinued early from the study (Fig. 1). The majority of subjects were female and white (*n*  =  136, 81.4% for both variables). The median age was 45.0 (19–64) years, and the mean body mass index was 28.7 (7.0) kg (Table 1). During the 6-month treatment period, 167 subjects had 2211 attacks, averaging 13.2 attacks per subject and 2.4 attacks per month. Those who completed the study (*n*  =  134) experienced 2036 attacks, averaging 15.2 attacks per subject and 2.5 attacks monthly.

**Table 1:** Demographics

Characteristic	Total (*N* = 167) n (%)
Sex	
Male	31 (18.6)
Female	136 (81.4)
Age (years)^a^	45.0 (19–64)
BMI (kg)^b^	28.7 (7.0)
Race	
American Indian or Alaska Native	0
Asian	2 (1.2)
Black or African American	25 (15.0)
Native Hawaiian or Other Pacific Islander	1 (0.6)
White	136 (81.4)
Other	3 (1.8)

*BMI* body mass index

^a^Median (range)

^b^Mean (SD)

### Medication usage

The median duration of DFN-02 exposure was 181 days. Over the course of the study, subjects treated 2190 attacks with 3292 doses of DFN-02, averaging 1.5 doses of DFN-02 per attack. An average of approximately one third (32%) of attacks per subject was treated with more than 1 dose of DFN-02. Each month, subjects took a mean of 3.6 DFN-02 doses, for a 6-month total of 19.7 doses; among those completing the study, the mean number of DFN-02 doses per month was 3.7, and the per-subject total was 22.6 (Table 2).

**Table 2:** Extent of DFN-02 exposure overall and in subjects who completed the study

	Safety population (*N* = 167)	Completers (*n* = 134)
Days^a^		
Mean (SD)	163.3 (48.9)	
Median	181	
Range	3–241	
Months^b^		
Mean (SD)	5.4 (1.6)	
Median	6.0	
Range	0.1–8.0	
Doses taken	3292	3031
Doses per patient		
Mean (SD)	19.7 (13.0)	22.6 (12.1)
Median	18.0	20.5
Range	(1–61)	(2–61)
Doses per patient (monthly)^c^		
Mean (SD)	3.6 (2.1)	3.7 (2.0)
Median	3.3	3.4
Range	0.3–10.2	0.3–10.2

*SD* standard deviation

^a^Date of study completion or early termination – date of enrollment +1

^b^Number of days in the study/30

^c^Number of total doses taken/number of months in the study

Most subjects (58%, 97/167) used rescue medication at least once in 6 months. The mean total doses per subject was 2.4 for 6 months, and the mean monthly average dose per subject was 0.5. The most commonly used rescue medications were ibuprofen (21%, 35/167); fixed combinations of aspirin-acetaminophen-caffeine (16.8%, 28/167) and aspirin-butalbital (5.4%, 9/167); and acetaminophen (5.4%, 9/167).

### Safety

In total, 71.9% (120/167) of subjects reported 1264 TEAEs in up to 6 months of treatment. About half of subjects who reported TEAEs (61/120) had mild TEAEs; only 9 had TEAEs at a level of discomfort that were determined by the investigator as severe. The severe TEAEs included dyspepsia, hiatus hernia, application site pain, gastroenteritis, diverticulitis, sinusitis, post-traumatic pain (worsening of pain from right shoulder injury noted in medical history), myalgia (transient entire body myalgia associated with each occurrence of study medication dosing), bladder injury (noted during an open hysterectomy procedure), and menometrorrhagia; each severe TEAE affected 1 subject, except for bladder injury and menometrorrhagia, both of which occurred in the same subject.

A total of 52.7% of subjects (88/167) experienced study medication-related events, with TEAEs in 35.9% of subjects (60/167) considered definitely related, in 11.4% of subjects (19/167) considered probably related, and in 5.4% of subjects (9/167) considered possibly related. One migraine TEAE and 3 headache TEAEs were considered related to study drug. Table 3 lists AEs related to treatment with DFN-02 that affected at least 2% of subjects.

**Table 3:** Treatment-emergent adverse events: total and those occurring in ≥2% of subjects by incidence, severity, and relationship to study medication^a^ (*N*  =  167)

	Subjects	Mild	Moderate	Severe	Not related	Possibly related	Probably related	Definitely related
Overall	120 (71.9)	61 (36.5)	50 (29.9)	9 (5.4)	32 (19.2)	9 (5.4)	19 (11.4)	60 (35.9)
Application site pain	51 (30.5)	38 (22.8)	12 (7.2)	1 (0.6)	0	2 (1.2)	9 (5.4)	40 (24.0)
Dysgeusia	35 (21.0)	30 (18.0)	5 (3.0)	0	0	4 (2.4)	7 (4.2)	24 (14.4)
Application site reaction	9 (5.4)	7 (4.2)	2 (1.2)	0	0	1 (0.6)	2 (1.2)	6 (3.6)
Application site irritation	7 (4.2)	7 (4.2)	0	0	0	1 (0.6)	0	6 (3.6)
Throat irritation	8 (4.8)	6 (3.6)	2 (1.2)	0	0	1 (0.6)	2 (1.2)	5 (3.0)
Chest discomfort	4 (2.4)	4 (2.4)	0	0	1 (0.6)	0	0	3 (1.8)
Nausea	7 (4.2)	2 (1.2)	5 (3.0)	0	1 (0.6)	1 (0.6)	2 (1.2)	3 (1.8)
Dizziness	6 (3.6)	4 (2.4)	2 (1.2)	0	0	3 (1.8)	1 (0.6)	2 (1.2)
Paresthesia	4 (2.4)	3 (1.8)	1 (0.6)	0	1 (0.6)	1 (0.6)	1 (0.6)	1 (0.6)
Vomiting	4 (2.4)	2 (1.2)	2 (1.2)	0	2 (1.2)	0	2 (1.2)	0
Diarrhea	4 (2.4)	3 (1.8)	1 (0.6)	0	3 (1.8)	0	1 (0.6)	0
Gastroenteritis viral	6 (3.6)	3 (1.8)	3 (1.8)	0	6 (3.6)	0	0	0
Influenza	4 (2.4)	1 (0.6)	3 (1.8)	0	4 (2.4)	0	0	0
Nasopharyngitis	12 (7.2)	10 (6.0)	2 (1.2)	0	12 (7.2)	0	0	0
Sinusitis	11 (6.6)	4 (2.4)	6 (3.6)	1 (0.6)	10 (6.0)	1 (0.6)	0	0
Upper respiratory tract infection	18 (10.8)	15 (9.0)	3 (1.8)	0	18 (10.8)	0	0	0
Urinary tract infection	5 (3.0)	3 (1.8)	2 (1.2)	0	5 (3.0)	0	0	0

^a^Values are n (%)

The most common TEAEs were application site pain, dysgeusia, application site reaction, upper respiratory tract infection, nasopharyngitis, and sinusitis (Table 3). Application site pain (including verbatim of nasal or nostril burning or stinging) was most common, affecting 30.5% of subjects (51/167) at least once over the 6-month study period; of those who reported application site pain, about three quarters (38/51) experienced mild pain, nearly one quarter (12/51) experienced moderate pain, and 1 subject experienced severe pain. The next most common TEAE, dysgeusia, was reported by 21% of subjects (35/167); the vast majority (30/35) of these subjects had mild dysgeusia, and 5 had moderate dysgeusia. Severe dysgeusia was not reported.

In the study, there were a total of 94 triptan-related TEAEs in 32 subjects (19.2%) (Table 3); three quarters of these subjects (24/32) had mild triptan-related TEAEs, and one quarter (8/32) had moderate events. Twelve subjects experienced triptan-related TEAEs that were considered by the investigator to be definitely related to study medication. An additional 12 subjects experienced triptan-related TEAEs that were considered probably related to study medication, 7 subjects experienced TEAEs that were considered possibly related to study medication, and 1 subject experienced a TEAE that was considered not related to study medication. The most common triptan-related TEAEs were dizziness and nausea (3.6%, 6 subjects each).

Four subjects experienced 5 SAEs; diverticulitis, cholecystitis, and menometrorrhagia each affected 1 subject, and a fourth had both pyelonephritis and myocardial infarction. Three SAEs were treatment-emergent (diverticulitis, pyelonephritis, and menometrorrhagia each affected 1 subject), but all 5 SAEs were considered not related to study medication.

Five subjects experienced a total of 10 TEAEs leading to discontinuation from study medication. These TEAEs included dizziness (1.2%), which affected 2 subjects, and diarrhea, dyspepsia, nausea, vomiting, feeling jittery, pain, lethargy, and dyspnea, each of which occurred in 1 subject (0.6%). Another subject was discontinued due to cholecystitis, an SAE requiring hospitalization, but it was not treatment-emergent and was considered not related to the study medication. There were no clinically meaningful trends in mean changes from baseline or individual changes for any clinical laboratory variable, vital sign, or ECG.

## Discussion

In this 6-month, multicenter, open-label safety study — during which study medication was used to treat more than 2000 migraine attacks — DFN-02 was safe and tolerable for the acute treatment of patients with episodic migraine with and without aura. Adverse events with DFN-02 were similar in pattern, frequency, and severity to those seen in previous research with triptans and nasally administered medications [17, 35], and no novel safety signals were seen. As expected, dysgeusia and application site pain were the most commonly reported tolerability issues; most TEAEs were mild, however, which may have been a factor in the high rate of study completion (77.5%).

Although there were no efficacy assessments completed in this study, findings on other measures indicate that subjects using DFN-02 appear to have experienced migraine relief. For example, the vast majority of subjects completed the study, treating multiple attacks over the 6-month study period, and rescue medication was only used in about 18% of attacks (2.4 doses for an average of 13 attacks). Moreover, although repeat dosing of DFN-02 was permitted at least 1 h after the initial dose, approximately two thirds of qualifying attacks were treated with a single dose of DFN-02. Since subjects were provided with multiple canisters of DFN-02 and could repeat the dose as needed to control their symptoms, the high proportion of attacks treated with a single dose suggests that DFN-02, which contains only 10 mg sumatriptan, provided most subjects with adequate migraine relief.

Previous research with the commercial formulation of intranasal sumatriptan 20 mg demonstrated good safety and tolerability; the incidence of individual AEs was similar to placebo [17, 35]. The persistent exception is bad, bitter, or unpleasant taste, which affected 19–36% of subjects in randomized, placebo-controlled studies [35] and 22%–27% of subjects in a pooled analysis [17]. With DFN-02, dysgeusia was similarly common (21% of subjects), but symptoms were mostly mild, and no subjects cited it as a reason for discontinuation. Application site pain with DFN-02 was more common than in previous research with intranasal formulations (30.5% vs 4% [17, 35]); these events may be related to the presence of the permeation enhancer DDM in DFN-02. In clinical practice, however, the kinetics of DFN-02 have been shown to be about the same as sumatriptan 4 mg SC [33], with migraine patients expected to experience similar times to onset of action and rates of pain freedom, which suggests that the risk of site reactions with DFN-02 could be about 30% lower than with comparable acute treatments, such as 4 mg SC (30.5% vs 43% [36]). Since most TEAEs were mild, the study completion rate was high, and few subjects withdrew, the relative frequency of application site pain does not seem a likely barrier to care with DFN-02.

This study provides clinically useful data but has some limitations. Subject eligibility was based on ICHD-2, while the current version is ICHD-3 (beta). At the time of the study set-up, ICHD-3 (beta) [1] was newly available and still collecting feedback from experts; therefore, the study proceeded using the previous version. Nevertheless, ICHD-2 and ICHD-3 (beta) are identical with respect to diagnosis of acute episodic migraine. Lacking a placebo control, the true incidence of AEs related to DFN-02 could not be determined. Additionally, in the absence of a direct comparison of DFN-02 with the commercially available intranasal formulation of sumatriptan, it was not possible to assess the relative safety of DFN-02 and marketed products. Given the well-established safety profile of intranasal sumatriptan, however, it is unlikely that any of these factors influenced the generalizability of these results or the long-term safety outcomes seen with DFN-02.

Sumatriptan is widely used and has been available in oral tablet, nasal spray, and injectable formulations for decades [25]. However, nearly 30% of migraineurs report dissatisfaction with acute therapies [37], with the most common complaint being that pain relief takes too long [22]; close to 90% of patients express a willingness to try new acute treatments [23, 24]. Because the PK of DFN-02 are compatible with a possible rapid onset of action and a favorable safety profile without the disadvantages associated with oral or SC therapies, DFN-02 may be useful for patients who prefer not to treat their condition with injectable medications, as well as patients in whom nonresponse, dysphagia, nausea, or vomiting preclude the use of orally administered drugs. As seen with similar triptan delivery systems [38, 39], ease of use may make DFN-02 especially convenient for treatment at home, work, and while traveling [35].

## Conclusions

In this multicenter, open-label safety study, DFN-02, a novel intranasal spray formulation composed of sumatriptan 10 mg and a permeation-enhancing excipient DDM, was safe and tolerable for the acute treatment of episodic migraine over a 6-month study. Adverse events with DFN-02 were similar in pattern, frequency, and severity to those seen in previous research with intranasal sumatriptan.

## Abbreviations

AE: Adverse event
DDM: 1-O-n-Dodecyl-β-D-Maltopyranoside
ECG: Electrocardiogram
ICHD: International Classification of Headache Disorders
IRB: Institutional Review Board
MedDRA: Medical Dictionary for Regulatory Activities
SAE: Serious adverse event
SC: Subcutaneous
SD: Standard deviation
TEAE: Treatment-emergent adverse event
